# Acetylcholine Promotes Ca^2+^and NO-Oscillations in Adipocytes Implicating Ca^2+^→NO→cGMP→cADP-ribose→Ca^2+^ Positive Feedback Loop - Modulatory Effects of Norepinephrine and Atrial Natriuretic Peptide

**DOI:** 10.1371/journal.pone.0063483

**Published:** 2013-05-16

**Authors:** Egor A. Turovsky, Mariya V. Turovskaya, Ludmila P. Dolgacheva, Valery P. Zinchenko, Vladimir V. Dynnik

**Affiliations:** 1 Department of Intracellular Signalling, Institute of Cell Biophysics, Russian Academy of Sciences, Pushchino, Russia; 2 Department of System Biochemistry, Institute of Theoretical and Experimental Biophysics, Russian Academy of Sciences, Pushchino, Russia; Medical University Innsbruck, Austria

## Abstract

**Purpose:**

This study investigated possible mechanisms of autoregulation of Ca^2+^ signalling pathways in adipocytes responsible for Ca^2+^ and NO oscillations and switching phenomena promoted by acetylcholine (ACh), norepinephrine (NE) and atrial natriuretic peptide (ANP).

**Methods:**

Fluorescent microscopy was used to detect changes in Ca^2+^ and NO in cultures of rodent white adipocytes. Agonists and inhibitors were applied to characterize the involvement of various enzymes and Ca^2+^-channels in Ca^2+^ signalling pathways.

**Results:**

ACh activating M_3_-muscarinic receptors and G_βγ_ protein dependent phosphatidylinositol 3 kinase induces Ca^2+^ and NO oscillations in adipocytes. At low concentrations of ACh which are insufficient to induce oscillations, NE or α1, α2-adrenergic agonists act by amplifying the effect of ACh to promote Ca^2+^ oscillations or switching phenomena. SNAP, 8-Br-cAMP, NAD and ANP may also produce similar set of dynamic regimes. These regimes arise from activation of the ryanodine receptor (RyR) with the implication of a long positive feedback loop (PFL): Ca^2+^→ NO→cGMP→cADPR→Ca^2+^, which determines periodic or steady operation of a short PFL based on Ca^2+^-induced Ca^2+^ release via RyR by generating cADPR, a coagonist of Ca^2+^ at the RyR. Interplay between these two loops may be responsible for the observed effects. Several other PFLs, based on activation of endothelial nitric oxide synthase or of protein kinase B by Ca^2+^-dependent kinases, may reinforce functioning of main PFL and enhance reliability. All observed regimes are independent of operation of the phospholipase C/Ca^2+^-signalling axis, which may be switched off due to negative feedback arising from phosphorylation of the inositol-3-phosphate receptor by protein kinase G.

**Conclusions:**

This study presents a kinetic model of Ca^2+^-signalling system operating in adipocytes and integrating signals from various agonists, which describes it as multivariable multi feedback network with a family of nested positive feedback.

## Introduction

The parasympathetic nervous system plays an important role in the control of circulating glucose and insulin [1]–[6]. Stimulation of parasympathetic nerves results in: acceleration of insulin production by pancreatic β-cells [5]–[7], suppression of glucose production and augmentation of glucose uptake by liver [4]. Acetylcholine (ACh), the principal neurotransmitter of the parasympathetic nervous system, realizes its metabolic effects by activating M_3_-cholinergic receptors (M_3_-AChR) in the pancreas [6]–[8], liver [9], [10], skeletal [11] and smooth [12] muscles and white adipose tissue (WAT) [13], [14]. In pancreatic [15], smooth [12], [16] and skeletal [17] muscle cells M_1,2_ AChR may be involved too.

Direct vagal (parasympathetic) control of WAT currently remains under debate [18]–[22], while the metabolic effects of ACh on glucose and lipid metabolism are not studied in details and some results are contradictory.

In prior studies, the metabolic effects of ACh have been characterized:

- by activation of glycogen synthesis by liver [9] and hepatocytes [9], [23], [24] and augmentation of glucose uptake by liver [25], [26] or conversely by activation of glyconeogenesis and glucose production by liver [9].
- by potentiation of glucose stimulated insulin production in β-cells [6], [7];
- by stimulation of glucose uptake in muscle cells [11], [17];
- by suppression of insulin stimulated glucose uptake in adipocytes [13].

The inhibitory effect of ACh on glucose uptake in WAT seems to be contradictory to its anabolic systemic insulin enhancing and glucose lowering effects. Moreover, in adipocytes activation of M_3_-mAChRs by ACh implicates the same signalling pathway as has been reported in pancreas [6]–[8]:

G_q_ proteins (G_q_)→Phospholipase C (PLC)→ diacylglycerol (DAG)/inositol-3-phosphate (IP3) → protein kinase C (PKC)/IP_3_receptor (IP_3_R)→Ca^2+^ (A).

On the contrary hepatic [27] and skeletal muscle [28] glucose uptake may be controlled by NO/cGMP/Protein kinase G (PKG) dependent signalling pathway and acceleration glucose uptake in skeletal muscles by Ach may also involve additional activation of calcium calmoduline dependent kinase kinase (CaMKK)/AMP kinase (AMPK) cascade [11].

Since the discovery of Ca^2+^ release from intracellular stores by IP_3_ and ACh [29] become widely recognized [27]–[31] that ACh promotes Ca^2+^-oscillations in various nonexcitable cells involving the PLC/IP_3_/Ca^2+^ dependent pathway (A) [30]–[34]. Yet, in different types of smooth muscle cells ACh may promote Ca^2+^-oscillations implicating either the classical PLC/IP_3_R signalling pathway (A) [31]–[33] or a NO/cGMP/PKG dependent pathway [12], [16], [35], [36], or combinations of both pathways [37]–[39].

The metabolic effects of Ca^2+^, the functional relevance of oscillatory regimes and the mechanisms of autoregulation of Ca^2+^-signalling machinery are not yet completely understood [30], [31], [34] and some results are contradictory. Furthermore, it remains unclear whether the diversity of the results obtained reflects the versatility of the signalling and metabolic networks used by the cells, or whether it corresponds to the controversy of results obtained.

Without doubt, different Ca^2+^-signalling pathways with multiple feedback and feedforward loops are implicated in the metabolic control of electrically nonexitable cells and may bring about complex of nonlinear dynamic behavior characterized by the set of temporal and spatial patterns usually registered as: Ca^2+^ elementary events (sparks), Ca^2+^-spikes, oscillations, spatial waves [30], [31], [37], coexistence of stable steady states (SST), simple and complex (multiperiodic and chaotic) oscillatory regimes and bifurcations (switching phenomena) [39]–[43].

The first example of Ca^2+^ oscillations was presented by Endo and coworkers in 1970 [44], when they discovered RyR encoded Ca^2+^ induced Ca^2+^ release (CICR) in skinned muscle fibers. Soon after, Ca^2+^ oscillations were registered in *Physarym P.* by Ridgway and Durham [45]. Later on Woods et al [46] demonstrated Ca^2+^ oscillations in non-excitable cells using single hepatocytes. Since that time various modes of Ca^2+^ sparks, oscillations and waves and switching phenomena have been registered in hepatocytes [39], [40], [46], pancreatic β-cells [6], [47] and acini [37], [38], [48], smooth muscle cells [12], [16], [32]–[35], adipocytes [49]–[51], pituitary cells [52], endothelial cells [53]–[55], astrocytes [57], fibroblasts [58], interstitial cells [56], and T-cells [59] in the presence of ACh, cholecystokinin (CCK), bradykinin (BK), norepinephrine (NE), phenilephrine, histamine, c-type natriuretic peptide, ATP, 8-Br-cGMP, NO-donors, NAD, cADP ribose (cADPR), L-arginine, L-glutamate, bovine fetal serum, etc. The wide variety of specific mechanisms may underlie the observed dynamic regimes.

This dynamic versatility is generally believed to involve oscillatory mechanisms based on: a short positive feedback loop (PFL) in the form of CICR via IP_3_R, with or without a modulatory role of the Gq→PLC→IP_3_R→Ca^2+^ axis [6], [30]–[34]; or an alternative oscillatory mechanism, arising from activation of PLC by Ca^2+^ and involving the long PFL Ca^2+^→PLC→IP3→IP_3_R→Ca^2+^ [6], [43], [60]–[62] or the inhibition of Ca^2+^-sensing receptors or G-proteins by PKC [63], [64].

Some authors have also considered tandem oscillatory mechanisms involving both types of CICR via IP_3_R and RyR [37]–[39], or suggested a modulatory role of the NO→cGMP→PKG axis in activation of PLC [48]. Only a few authors acknowledge the important role of CICR via RyR, with modulation by cADPR, in the generation of Ca^2+^ oscillations and waves in response to ACh or CCK [12], [16], [35], [36].

This focus of interest in IP_3_R has been mirrored in mathematical modeling. Most models describe various feedback mechanisms related to IP_3_R and PLC [41], [65]–[74]. Only few authors consider CICR via RyR as a possible oscillatory mechanism [75].

Alternative oscillatory mechanism, driving the propagation of NO and Ca^2+^ waves in colonic interstitial and smooth muscle cells was proposed in 1993 [56]. This mechanism included Ca^2+^→NO→Ca^2+^ positive feedback, which was based on known dependence of NO synthesis by constitutive NO synthase on Ca^2+^ [76], [77] and on observed mutual (reciprocal) amplification Ca^2+^ and NO signalling in interstitial cells [56].

Irrespective of Ca^2+^-oscillatory mechanisms, a very interesting kinetic model linking the NO→cGMP→PKG and cADPR→RyR→Ca^2+^ signalling pathways was offered some years later [78]. This model may be considered as a core mechanism of Ca^2+^ signalling via RyR in non-excitable cells. Taking all this into consideration, the main goal of this work is to show that ACh induces Ca^2+^ oscillations implicating both: Ca^2+^→NO→ cGMP→cADPR→Ca^2+^ long PFL and a short PFL based on CICR via RyR, with the possible involvement of other reinforcing PFLs. We additionally consider the modulator role of NE and of atrial natriuretic peptide (ANP) on this basic mechanism.

## Materials and Methods

### Isolation of Preadipocytes

All studies were approved by the Animal Ethic committee of the Institute of theoretical and experimental biophysics. NMRI mice (aged 3–5 weeks) were decapitated after a brief (45–60 sec) anesthesia with carbon dioxide before sacrifice. Mice were subjected to cervical dislocation and disinfected with 70% ethanol prior to dissection. All operations were performed in a sterile environment on ice. White adipose tissue was removed from the epididymal fat depot and placed in a Petri dish with cold DMEM medium (Sigma, USA). Scissor-minced white adipose tissue was transferred into a tube containing sterile DMEM with 7 mg collagenase II (Sigma) and 4% bovine serum albumin (BSA, free from fatty acids). Then the tissue was incubated for 18 min at 37°C. To stop the enzymatic reaction, the tube was chilled on ice for 20 min with intermittent shaking followed by filtration through 250 µm filter and centrifugation at 1000 g for 10 min. The pellet was then resuspended in cold DMEM medium, filtered through 50 µm filters and centrifuged at 1000 g for 10 min. Finally, the pellet was resuspended in cultural medium containing: DMEM (Sigma), 10% fetal bovine serum (FBS; Gibco), 4 mM *L*-glutamine, 4 nM insulin, 0.004% gentamicin and 25 µg/ml sodium ascorbate (Sigma). The obtained suspension contained preadipocytes, since mature adipocytes carry vesicles of fat and do not precipitate under the given conditions.

### Cultures of Preadipocytes and Adipocytes

100 µl droplets of culture medium containing 3×10^4^ preadipocytes were placed on round coverglasses (25 mm in diameter), which were then transferred into 35 mm Petri dishes. 6 hours after adhesion of the cells to the glass, additional culture medium was added to the Petri dishes. On the third day the medium in the dishes was replaced with a fresh portion of medium, which included 10 nM cytosine arabinoside (Sigma) to suppress the proliferation of fibroblasts, and incubation in CO_2_ atmosphere was continued for 8 hours. After that the medium was replaced with fresh culture medium. On the ninth day of culture, the cells form a monolayer and become differentiated. An important morphological difference of mature white adipocytes from other cells is formation of fat droplets (Supplemental, Fig. S1A).

### The Measurement of Cytosolic Calcium Concentration ([Ca^2+^]_i_)

The measurement of [Ca^2+^]_i_ was performed by fluorescent microscopy using Fura-2/am (Molecular probes, USA), a ratiometric fluorescent calcium indicator (Supplemental, Fig. S1B). Cells were loaded with the probe dissolved in Hanks balanced salt solution (HBSS), containing 10 mM HEPES and 200 µM L-arginine, pH 7.4, at a final concentration of 5 µM at 37°C for 40 min with subsequent 15 min washout. The coverslip containing the cells loaded with Fura-2 was then mounted in the experimental chamber. During the experiment we used a home-made perfusion system, which enables complete replacement of the cell bathing solution within 30 seconds. We used an Axiovert 200M based imaging system (Carl Zeiss, Germany) equipped with HBO100 mercury lamp, AxioCam HSm CCD camera and MAC5000 high speed excitation filter wheel. Fura-2 fluorescence was excited at two wavelengths using band-pass filters BP 340/30 and BP 387/15; fluorescence was registered in the wavelength range of 465–555 nm. Excitation light intensity was lowered using 25 and 5% neutral density filters in order to prevent phototoxicity. Image frames were acquired at 3 s intervals with a Plan Neofluar 10×/0.3 objective. The time lapse image sequences were analyzed using ImageJ 1.44 (NIH Image, Bethesda, MD). Graphs were plotted using OriginPro 8.0 software.

### Measurements of NO Production and [Ca^2+^]_i_

NO production in white adipocytes was evaluated using the fluorescent probe DAF-FM diacetate (Molecular Probes, USA), oxidation of which by NO dramatically increases the quantum yield of the dye [1]. Cells were loaded with 5 µM DAF-FM for 40 min at 37°C. For simultaneous monitoring of intracellular NO and Ca^2+^, 4 µM Fura-2AM was added to the medium. After loading, the cells were additionally incubated in Hanks balanced salt solution (HBSS) for 20 min to complete the deesterification of the dyes.

Dye-loaded cells were visualized using a Cell Observer imaging system (Carl Zeiss, Germany). DAF-FM fluorescence was excited using a BP 475/40 filter. BP 340/30 and BP 387/15 filters were used to excite the Ca^2+^-bound and Ca^2+^-free forms of Fura-2, respectively. The emission of both DAF-FM and Fura-2 was recorded at 530±25 nm. In the case of double staining (Fura-2 and DAF-FM), the total exposure time per three-channel frame was 20 s. Collected 8-bit time-lapse images were analyzed using ImageJ software with Time Series Analyzer and RatioPlus plugins.

The changes in [Ca^2+^]_i_ are presented as the 340/380 ratio obtained from time-lapse images after background subtraction. DAF-FM fluorescence is presented as ΔF/F_0_, where ΔF = F – F_0_ (F is the mean fluorescence of DAF-FM, and F_0_ is the initial fluorescence). The experimentally obtained curves were smoothed to decrease the effect of noises. The curve of ΔF/F_0_ characterizes the fluorescence intensity of benzotriazole, a product of nitrosylation of DAF-FM accumulated in the cells. For a more precise estimation of the changes in NO concentration, the derivative d(ΔF/F_0_)/dt was used, assuming that the rate of benzotriazole dissociation is very slow in comparison with life time of free NO and therefore NO concentration is proportional to the derivative d(ΔF/F_0_)/dt. All imaging experiments were performed at room temperature (25–26°C).

During all the stages of probe loading and washout, as well as during the application of various agonists, the HBSS medium contained 200 µM L-arginine to maintain the active state of eNOS, and cGMP metabolizing enzymes [14]. In the absence of L-arginine basal NO production was detected only in single adipocytes.

### Statistical Analysis

The experiments were performed at least 3–5 repeats for 2–3 cell cultures of adipocytes. N – represents the number of cells in the experiments. Statistical analysis was performed using Origin 8 (Microcal Software Inc., Northampton, MA) software. Results are expressed as means ± standard deviation (SD). Differences were deemed to be significant at p<0.05.

## Results

### [Ca^2+^]i Oscillations Induced by ACh. The Role of M_3_-cholinergic Receptors and G-proteins

**Figure 1:**
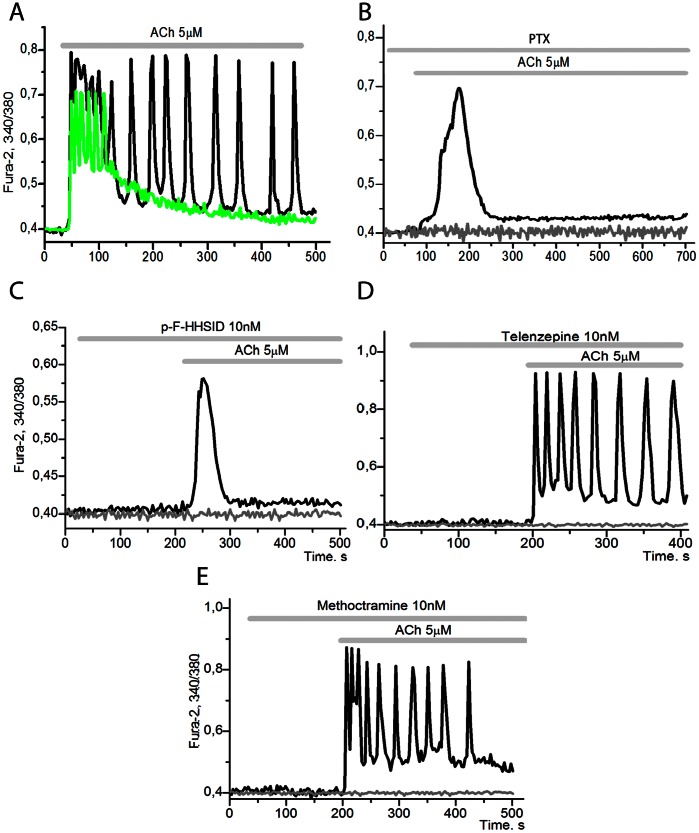
The changes of [Ca^2+^]_i_ in adipocytes upon the effect of 5 µM ACh and inhibitors of mAChR. A: 5 µM acetylcholine induces oscillations of cytosolic calcium in white adipocytes, as measured by Fura-2 ratio. Here and in later experiments the responses of individual adipocytes are presented. Number of cells in the experiments (n) = 253. **B**: adipocytes cultured for 24 hours in the presence of 100 ng/ml of pertussis toxin (PTX) are not capable of generating [Ca^2+^]_i_ oscillations. 92±3,4% of the cells the response to ACh application is absent (grey curve), and the transient signal (black curve) is observed in 8±2,3% of the cells (n = 128). **C**: in the presence of 10 nM pF-HHSID there is no response to ACh in 90±2,8% of the cells, and the transient signal (black curve) is observed in 10±3,2% of the cells, n = 324. **D**: in the presence of 10 nM Telenzepine ACh causes [Ca^2+^]_i_ oscillations in 88±9,4% of adipocytes, while in 13±1,5% of adipocytes the oscillations are absent, n = 8. **E**: in the presence of 10 nM Methoctramine hydrate the oscillations are observed in 89±3,8% of adipocytes (black curve), while 15±14,8% of the cells give no response, n = 200.

Figure 1A shows a typical example of Ca^2+^ oscillations induced by ACh. ACh in the concentration range of 500 nM –10 µM can provoke two types of response: a series (trains) of Ca^2+^ impulses (Fig. 1A, green curve) in 72±07% of the cells, and sustained Ca^2+^ oscillations in 16±4,6% of the cells (Fig. 1A, black curve). Low concentrations of ACh (up to 50 nM) do not produce any changes of [Ca^2+^]_i_.

The aforementioned periodic modes (Fig. 1A) disappear after incubation of the cells for 24 hrs with 100 ng/ml of pertussis toxin (PTX), a blocker of G_i_-proteins (Fig. 1B). Notably, up to 92±3,4% of the cells do not respond upon addition of ACh, and the transient signal is observed in only 8±2,3% of the cells (black curve). Oscillations also disappear in the presence of M_3_–cholinergic receptor antagonist, pF-HHSID (Fig. 1C) and could not be altered by pretreatment of adipocytes with the antagonists of M_1_- and M_2_-cholinergic receptors, 10 nM telenzepine (Fig. 1D) or 10 nM methoctramine hydrate (Fig. 1E). Thus the data presented on Fig. 1 indicate that ACh induces Ca^2+^ -oscillations through activation of M_3_-cholinergic receptors and heterotrimeric G-proteins.

### PLC and IP_3_R do not Participate in Generation of Ca^2+^ Oscillations Induced by ACh

**Figure 2:**
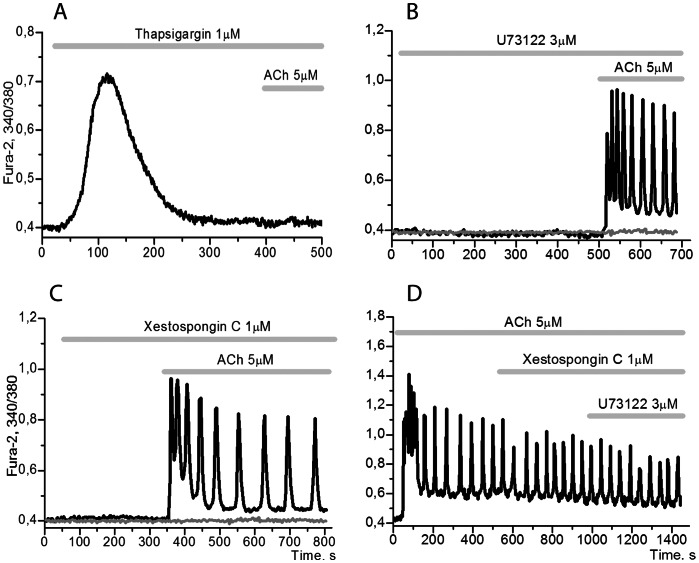
Role of reticular Ca^2+^ and PLC/IP_3_R signalling axis in Ca^2+^oscillations. **A**: in the presence of 1 µM inhibitor of SERCA, Thapsigargin, 100% adipocytes do not respond to ACh (n = 160). **B**: in the presence of 3 µM U73122 the oscillations are observed in 77±10,2% of adipocytes (n = 152). **C**: in the presence of 1 µM Xestospongin C the oscillations are observed in 80±6,7% of adipocytes (n = 259). **D**: simultaneous incubation of the cells with 1 µM Xestospongin C and 3 µM U73122 does not suppress [Ca^2+^]_i_ oscillations in 18±4,1% of adipocytes, which responded with sustained modes of oscillations to ACh (n = 165).

The steady modes of Ca^2+^ oscillations observed in adipocytes persist during a short period of time (about 3–4 minutes) in the absence of Ca^2+^ in the incubation medium [14] and disappear completely upon depletion of the reticular Ca^2+^ pool following incubation of cells with thapsigargin, an inhibitor of the Ca^2+^-ATPase of endoplasmic reticulum (SERCA) (Fig. 2A). This indicates that the entry of Ca^2+^ from the extracellular medium into the cell in the context of active extrusion of [Ca^2+^]_i_ by plasma membrane Ca^2+^-ATPase (PMCA) is a prerequisite for maintaining the oscillations, but does not have any significant role in the mechanisms underlying generation of the oscillations.

Preincubation of adipocytes with inhibitors of PLC, U73122 (Fig. 2B) or of IP_3_R, Xestospongine C (Fig. 2C) had no influence on Ca^2+^ oscillations induced by ACh in most cells responding either by trains or by steady modes of Ca^2+^ oscillations.

Furthermore, monitoring those cells with steady modes of Ca^2+^ oscillations, we found that application of both inhibitors simultaneously also had no effect on the observed responses (Fig. 2D). The frequencies and the amplitudes of Ca^2+^ oscillations remain within the limits of characteristic values for ACh. The same is also true for PKC inhibitors (not shown).

These results indicate that the classical mechanisms of Ca^2+^ oscillations, the PLC dependent Ca^2+^-signalling pathway (A), the long PFL based on activation of PLC by Ca^2+^, and CICR via IP_3_R do not operate under the conditions of the aforementioned experiments. However, these mechanisms may still be realized in adipocytes, where they are responsible for the generation of a so called “baseline” Ca^2+^ oscillation observed in the presence of fetal bovine serum [50], [51]. It is known that activation of IP_3_R and PLC can be controlled by phosphorylation of the adaptor protein IRAG [79]–[82] and of the protein Regulators of G-protein Signaling (RGS) [83], [84] respectively by PKG. Such phosphorylation resulting in inhibition of IP_3_R and PLC may correspond to the formation of negative feedback loops (NFLs) under the control of PKG, which could be responsible for switching off Ca^2+^-signalling pathway (A) under the conditions used in our experiments.

### ACh Activates the Gβγ→PI3Kγ→PKB→eNOS Signalling Axis

**Figure 3:**
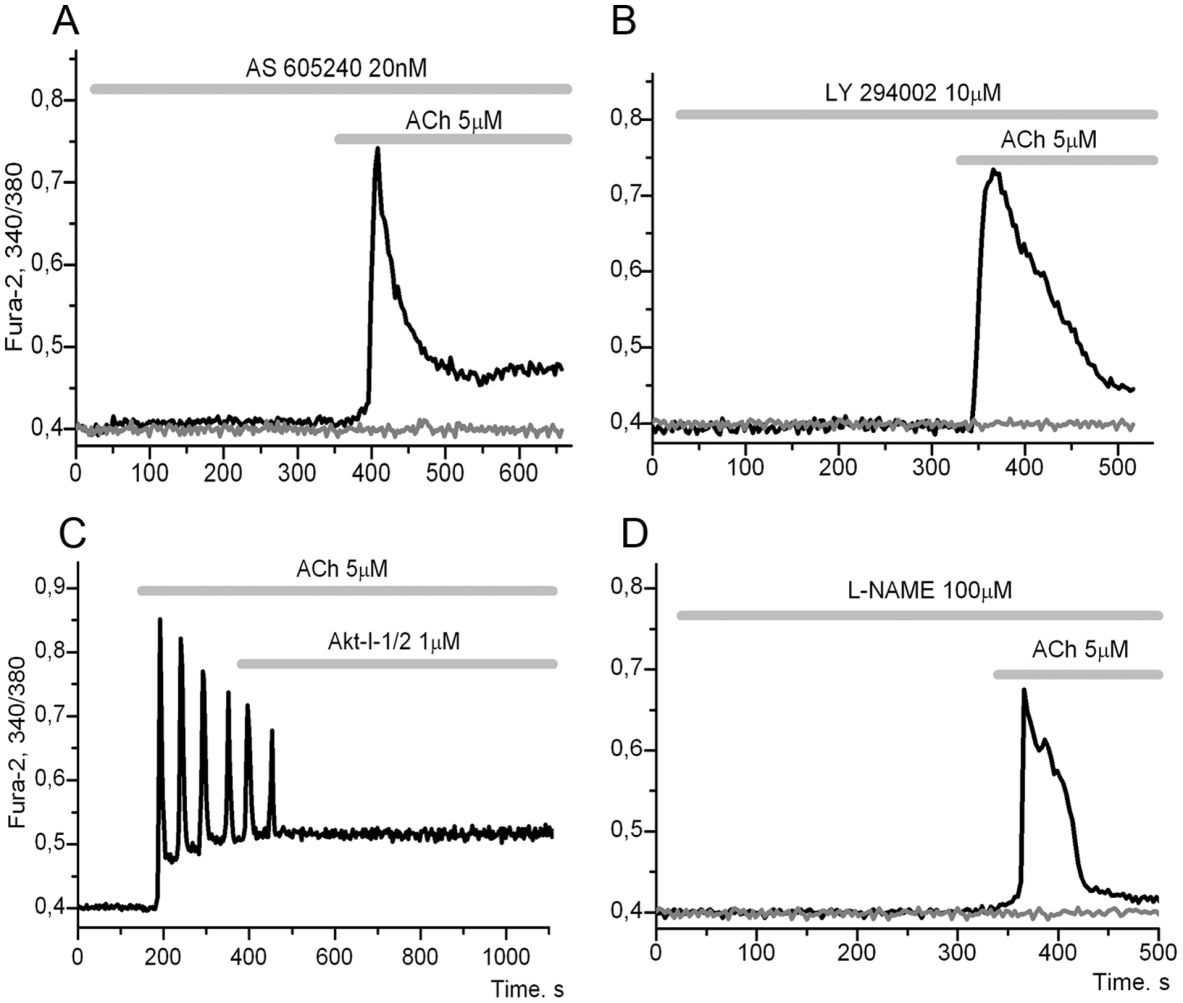
Involvement of PI3Kγ/PKB/eNOS in oscillations induced by ACh. **A**: [Ca^2+^]_i_ response to 5 µM ACh is absent in 84±9,4% of cells in presence 20 nM AS-605290 (PI3K inhibitor) and [Ca^2+^]_i_ transitory response was recorded in 21±4,9% of cells, (n = 260). **B**: [Ca^2+^]_i_ response to 5 µM ACh is absent in 89±7,9% of cells in the presence 10 µM LY-294,002 (PI3K inhibitor), while [Ca^2+^]_i_ transitory response was recorded in 13±7,4% of cells, (n = 145). **C**: [Ca^2+^]_i_ oscillations were inhibited by AKT1/2 (PKB inhibitor) in 68±5,5% cells, (n = 168). **D**: [Ca^2+^]_i_ response to 5 µM ACh is absent in 82±8,4% of cells in presence of 1 µM L-NAME (eNOS inhibitor) and [Ca^2+^]_i_ transitory response was recorded in 18±6,3% of cells, (n = 6).

Preincubation of adipocytes with the selective phosphoinositide 3-kinase gamma (PI3Kγ) inhibitor AS-605290 (Fig. 3A) or with the nonselective PI3K inhibitor LY-294002 (Fig. 3B) resulted in the suppression of oscillatory modes induced by application of ACh, similar to those results shown with incubation of cells with PTX and p-F-HHSID (Fig. 1B, C).

Only a small proportion of the cells retained an impulse-shaped Ca^2+^ response, which may be the result of activation of the G_αq_→PLC→IP_3_→ IP_3_R→Ca^2+^ signalling pathway (A) under these conditions. It is known that class 1A PI3Kγ is activated by the G protein beta gamma subunits of G proteins (G_βγ_-subunits) of G_q_ and G_i_ proteins [85], [86]. And endothelial NO synthase (eNOS) is phosphorylated and activated by PKB [87], [88]. This means that the effects of inhibitors of PI3K (Fig. 3A, B), protein kinase B (PKB) (Fig. 3C) or eNOS (Fig. 3D), as well as inhibitory effect of PTX (Fig. 1B), may result from blocking the signalling axis:

G_βγ_→PI3Kγ→PKB→eNOS.

Functioning of this signalling axis has previously been demonstrated in endothelial cells under the action of endothelin-1 [89].

### Ca^2+^ Signalling Pathways Convergence with the Involvement of ACh and Norepinephrine or α-adrenergic Agonists

**Figure 4:**
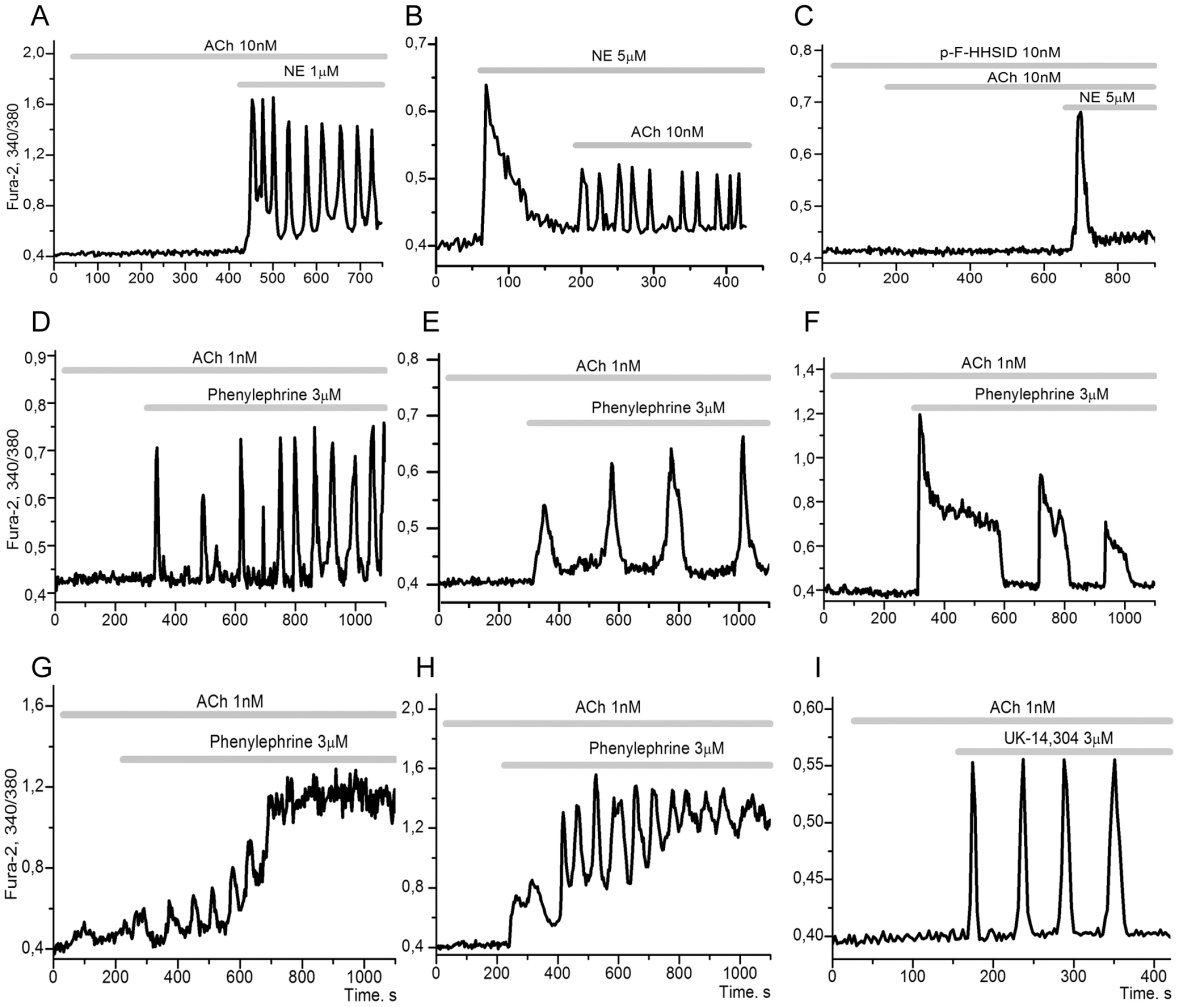
The potentiation of [Ca^2+^]_i_ oscillations by α-adrenergic agonists. **A**: upon the application of NE (1 µM) in the presence of 10 nM ACh [Ca^2+^]_i_ the onset of oscillations is observed in 55±5,8% of adipocytes, (n = 144). **B**: upon the application of 10 nM ACh in the presence of 5 µM NE the oscillations arise in 47±6,8% of the cells, all the rest showing either no response or a transient increase in [Ca^2+^]_i_, (n = 110). **C**: upon the application of NE (5 µM) in the presence of 10 nM M_3_-cholinergic receptor antagonist (p-F-HHSID) and 10 nM ACh, the transient increase of [Ca^2+^]_i_ is observed in 73±8,0% of adipocytes, with no periodic modes of Ca^2+^ detected, (n = 150). The transient increase of calcium upon NE application is a classic demonstration of adrenergic receptor activation. **D**: the onset of fast Ca^2+^-oscillations in 37±5,4% of the cells upon application of 3 µM selective agonist of α_1_-adrenergic receptors, Phenylephrine, (n = 185). **E**: the generation of impulse shaped oscillations in 24±4,4% of the cells upon application of 3 µM Phenylephrine, (n = 115). **F**: the onset of relaxation type oscillations with long period in 08±3,4% of the cells, (n = 167). **G**: upon the application of 3 µM Phenylephrine against 1 nM ACh the Ca^2+^ signalling system switches to a new steady state with high values of [Ca^2+^]_i_ n 07±2,1% of adipocytes, (n = 147). **H**: the transition to a new steady state with high mean values of [Ca^2+^]_i_ for the oscillation period occurs in single adipocytes, (n = 18). **I**: the generation of impulse [Ca^2+^]_i_ oscillations in 24±5,4% of the cells upon the application of 3 µM selective agonist of α_2_-adrenergic receptor, UK-14,304, against 1 nM Ach, (n = 138). In the absence of ACh (control experiment), upon the application of UK-14,304 adipocytes respond with a single impulse increase of [Ca^2+^]_i_, but they never respond with oscillations.

In the presence of low concentrations of ACh (less than 50 nM), which cannot induce Ca^2+^ responses in adipocytes, application of 1–10 µM norepinephrine (NE) lead to the generation of steady periodic modes in 55±5,8% of the cells (Fig. 4A) whereas addition of NE alone in the same concentration range induced either impulse responses in the majority of cells, or fast-damping oscillations in single adipocytes [14]. The addition of low doses of ACh after application of NE also resulted in generation of sustained modes of Ca^2+^ oscillations (Fig. 4B). The observed effects of synergistic action of ACh and NE persisted in the presence of propranolol, an inhibitor of β-adrenergic receptors (not shown) but disappeared in the presence of p-F-HHSID, an inhibitor of M_3_-cholinergic receptors (Fig. 4C).

**Figure 5:**
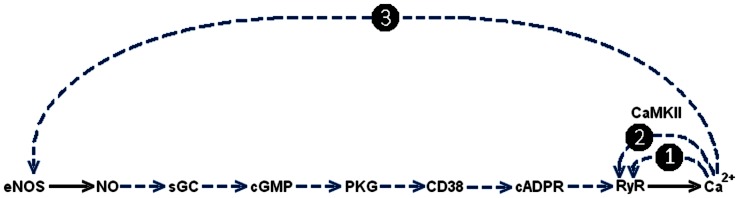
Minimal list of PFLs operating in Ca^2+^-signalling system (B). Dasher blue numbered arrows indicate various PFLs appearing in the system and formed by Ca^2+^ induced Ca^2+^ release by RyR (1), phosphorylation of RyR by CaMKII (2) and phosphorylation of eNOS by Ca^2+^(3). For details see the text.

The steady periodic or quasi periodic modes of Ca^2+^-oscillations are also observed upon the addition of an α_1_-adrenergic receptor selective agonist, phenylephrine (Fig. 5D–H), or an agonist of the α_2_- adrenergic receptor, UK-14340 (Fig. 4J). These agonists also do not generate sustained oscillations of [Ca^2+^]_i_ in the absence of ACh in the presented concentration range [14]. Unlike the effect of NE, which induces generations of simple modes of fast Ca^2+^-oscillations in the presence of ACh (Fig. 5A, B), the agonists of α_1_-, α_2_-adrenergic receptors can result in appearance of various types of dynamic behavior in the Ca^2+^-signalling system. Synergic action of ACh and α-adrenoreceptor agonists can create complex nonlinear dynamic behavior, including:

- generation of “classic” fast Ca^2+^ oscillations with a period of several dozens of seconds (Fig. 4D);
- switching of the Ca^2+^ signalling system to a new stable steady state (SST) with higher values of [Ca^2+^]_i_ (Fig. 4G);
- transition to a new steady quasiperiodic mode of oscillations with high mean values of [Ca^2+^]_i_ for the oscillation period (Fig. 4H);
- generation of impulse-shaped (Fig. 4E, I) and relaxation-type Ca^2+^ oscillations with a long unfixed periods (Fig. 4F).

Such diversity of dynamic behavior in the Ca^2+^ signalling system was observed even within a single culture of cells (9 DIV) and was detected in adipocytes for the first time. No doubt the cells in culture have different sets of parameters (activities of enzymes and channels), being in different stages of their development. This parametric diversity may underlie the versatility of dynamic behaviour observed, although we cannot exclude the possibility that different cells with similar sets of parameters have different concentration ranges for key metabolites (Ca^2+^, NO, cGMP, cADPR, etc). In both cases application of a second agonist promotes various periodic modes or switching phenomena.

Previously, using inhibitory analysis [14] we have shown that two types of response: the generation of Ca^2+^ oscillations in adipocytes promoted by ACh and the Ca^2+^ impulses arising from the effect of NE or α_2_-adrenergic receptor agonists - UK-14,304, guanabenz and L-arginine, can be realized with the implication of the following signalling pathway:

PI3K→PKB→eNOS→sGC→PKG→CD38→RyR→ Ca^2+^ (B)

The induction of Ca^2+^ oscillations and switching phenomena promoted by NE or by α-adrenoreceptor agonists in the presence of low concentrations of ACh (Fig. 4) provide evidence for convergence of Ca^2+^ signalling pathways with the involvement of M_3_AcR and α_1_- and α_2_-adrenergic receptors at the level of G_βγ_-subunits of G_q_ and G_i_-proteins. ACh signal enhancement by α-adrenergic agonists, via G_βγ_-proteins (activating PI3Kγ), may thus be responsible for the synergic activation of signalling pathway (B) and observed dynamic versatility.

### Possible Interaction of PLC- and eNOS-dependent Pathways

Taking into consideration those G_βγ_-proteins may additionally activate PLC_β_ and PLC_δ_ [90], [91], it is possible that activation of M_3_-AChRs and of α_1_- α_2_-adrenoreceptors switches on both Ca^2+^ signalling pathways implicating IP_3_R and RyR:

G_αq_, G_βγ_→PLCβ→ IP_3_→IP_3_R→Ca^2+^ (A)

G_βγ_→PI3Kγ→ PKB→ eNOS→ NO→ sGC→ cGMP→ PKG→ cADPR→RyR→Ca^2+^ (B)

The impact of the PLC signalling pathway on Ca^2+^ oscillations induced by ACh may depend on the extent of IP_3_R inhibition due to phosphorylation of IRAG protein by PKG [79]–[82] and on activity of PLC, which may be controlled by phosphorylation of RGS proteins by PKG [83], [84].

In turn, PKG activity may strongly depend on the presence of L-arginine in incubation medium [14]. It is known that activation of PKG and PDEV is a relatively slow process [92]. Most prior experiments on isolated cells of different types have been performed using mineral incubation media devoid of amino acids, with the expectation that cells preserve enough of amino acids during experiments. However, under the leak of L-arginine from the cells, especially in the presence of dimethyl arginine, an inhibitor of eNOS [93], we would expect under-activation of the signalling axis (B) and a dominating role of the signalling axis (A) Taking all this into consideration we used incubation media containing L-arginine in all experiments.

Thus, the results presented above indicate that in the presence of low concentrations of L-arginine in medium (100–200 µM), ACh and NE potentiate Ca^2+^-signalling pathway (B) through activation of M_3_-mAChR (G_q_) and α1 (G_q_) and α2 (G_i_) adrenoreceptors respectively. Active functioning of this pathway may be responsible for the complex nonlinear dynamic behaviour observed (Fig. 4) and for the switching off Ca^2+^-signalling pathway (A) (Fig. 2).

### Short and Long PFLs in the Ca^2+^ Signalling System (B)

The mechanism of Ca^2+^-induced Ca^2+^-release via RyR [44], [94] may create the first PFL in the system, hereafter termed short PFL(1):

Ca^2+^→ RyR→ Ca^2+^ (1)

The known activation of RyR by Ca^2+^ dependent calmodulin kinase (CaMKII) [95] may also bring about a second short PFL (2):

Ca^2+^→CaMKII→RyR→Ca^2+^ (2)

This short PFL(2) may reinforce the functioning of short PFL(1).

It is well known also that NO synthesis by constitutive NO synthases is strongly Ca^2+^ dependent [76], [77], and vice versa NO, cGMP, cADPR and βNAD can all activate Ca^2+^ release via RyR [96]–[99]. This allows the possibility of functioning of Ca^2+^→NO→Ca^2+^ positive feedback loop, as was predicted 20 years ago [56].

This long feedback loop arising in Ca^2+^ signalling pathway (B) represent long PFL(3):

Ca^2+^→eNOS→NO→sGC→cGMP→PKG→CD38→cADPR→RyR→ Ca^2+^ (3)

All these loops are shown in figure 5 and may be considered as a family of nested loops, where long PFL(3) embraces both short PFLs(1,2). This long PFL(3) acts as a generator of cADPR, a coagonist of Ca^2+^ which potentiates activation of RyR. Active interplay of these loops may produce various types of Ca^2+^ oscillations and other non-linear dynamic effects observed (Fig. 4). Of course this is minimal list of PFLs with may operate in the system.

### The Role of eNOS and PKG in the Oscillatory Mechanisms

**Figure 6:**
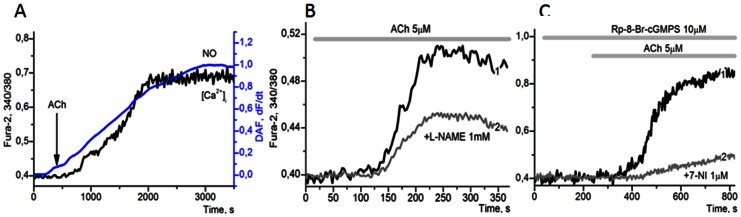
Breakdown of long PFL by eNOS and PKG inhibitors. **A**: the changes in [Ca^2+^]_i_ level (black curve) and NO accumulation in DAF-FM (ΔF/F_0_, blue curve) upon the application of 5 µM ACh after cell preincubation within 5 minutes with 5 µM inhibitor of PKG, KT5823, in 37±8,6% of cells, (n = 93). **B**: the effect of of PKG inhibitor, KT5823 (5 µM; curve 1), and KT5823+ inhibitor of NOS, L-NAME (1 mM; curve 2) added 5 minutes before the start of calcium dynamics registration in the cytosol, (n = 137). Slow increase of [Ca^2+^]_i_ occurs in 51±19,4% of the cells (curve 1) and in 35±8,0% of cells (curve 2). **C**: the effect of PKG inhibitor, Rp-8-Br-cGMPS, (10 µM) which prevents the onset of periodic modes in all cells and results in a rise of Ca^2+^ in the cytosol in 30±4,5% of adipocytes (curve 1). The application of 5 µM ACh against a background of Rp-8-Br-cGMPS and NOS inhibitor, 7-NI (1 µM) leads to slow rise of Ca^2+^ in the cytosol in 11±3,2% of adipocytes (curve 2), n = 214.

The functioning of long PFL(3) may be prevented by inhibitors of any enzyme in this loop. Preincubation of cells with the PKG inhibitors KT5823 and Rp-8-Br-cGMPS not only leads to the suppression of the observed oscillatory modes in 92±5,4% (±SD) of the cells, but also results, after a short lag phase, in a slow increase of [Ca^2+^]_i_ and NO (Fig. 6A) in 37±8,6% of the cells. Upon preliminary addition of inhibitors of eNOS, L-NAME or 7-NI this slow increase of [Ca^2+^]_i_ is either completely suppressed in half of the cells, or is significantly decreased in 35±8,0% of the cells (Fig. 6B, C – curves 2). The observed increase of [Ca^2+^]_i_ upon PKG inhibition might be related to:

- activation of the signalling pathway (A) due to the reversal of the inhibitory effect of PKG on IP_3_R and PLC,
- nitrosylation of reticular Ca^2+^ channels by NO,
- decreased activity of Ca^2+^-ATPases under these conditions, since PKG-dependent phosphorylation may result in activation of the Ca^2+^-ATPases SERCA and PMCA. Indirect activation of both these enzymes via phosphorylation of phospholamban (for SERCA) [100], [101] and PDZ domain (for PMCA) [102], [103] by PKG may correspond to feedforward activation mechanisms, dynamically equivalent to operation of NFLs.

Suppression of the Ca^2+^- release process by the combined action of inhibitors of eNOS and PKG (Fig. 6B, C; curves 2) indicate an important role of NO/PKG in the operation of long PFL(3) and in control of Ca^2+^-ATPases.

Thus, the obtained data confirm the important role of Ca^2+^ in activation of eNOS leading to accumulation of NO and, vice versa, indicate the important role of NO and PKG in the control of [Ca^2+^]_i_ level.

### The Shapes and Phase Relationships between Ca^2+^ and NO Oscillations

Owing to the short lifespan of free NO and the slow dissociation kinetics of DAF*NO complexes (of benzotriazole), the recorded value of ΔF/F_0_ represents an integral of NO concentration accumulated by DAF. The derivative of ΔF/F_0_ (value d(ΔF/F_0_)/dt) may thus be used as an estimate of free NO levels. In this manner we can monitor NO oscillations in cells undergoing long period Ca^2+^ oscillations.

From a mathematical point of view, considering [Ca^2+^]_i_ as a fast variable of the autocatalytic mechanism, we should expect that a slow variable or variables must also be present within this long PFL. For example, this may be NO or processes linked to NO production (i.e. eNOS or PKG activities).

**Figure 7:**
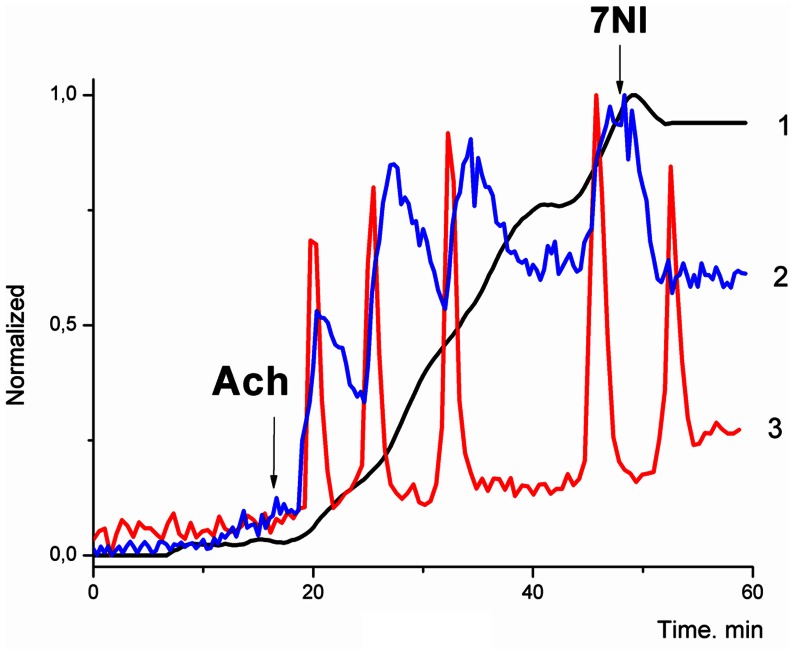
The regimes of slow Ca^2+^ and NO oscillations induced by ACh. The black curve (1) indicate integral NO level, accumulated by DAF (ΔF/F_0_), blue curve (2) corresponds to NO (d(ΔF/F_0_)/dt), the red curve (3) indicate changes in [Ca^2+^]_i_.

The slow Ca^2+^ and NO oscillatory mode with a period of several minutes (Fig. 7) confirms an important role of long PFL(3) in the generation of stable periodic regimes in the cells. In the first approximation, analyzing the shapes and phase relationships between [Ca^2+^]_i_ (Fig. 7, black curve) and NO (Fig. 7, d(ΔF/F_0_)/dt, green curve), we may consider [Ca^2+^]_i_ as a fast variable and NO (or a process related to NO production) as a slow variable in the oscillatory mechanism. The rise in [Ca^2+^]_i_ level is followed by an increase in NO production which may indicate the direct activation of the eNOS by Ca^2+^. The maximal values of NO correspond to the minimal values of [Ca^2+^]_i_. This may reflect the acceleration of [Ca^2+^]_i_ extrusion by Ca^2+^ ATPases SERCA and PMCA, owing to their possible activation due to phosphorylation by PKG.

The impulse shape of [Ca^2+^]_i_ oscillations and the sawtooth shape of NO oscillations also indicate that Ca^2+^ and NO can be considered as the fast and slow variables of this oscillatory mechanism.

A phase shift between the maxima of Ca^2+^ and NO of about 30 sec or more may indicate that besides fast activation of eNOS by Ca^2+^ (by CaCaM [76], [77]), some slower activation of eNOS by any Ca^2+^-dependent kinases operating in the system may also occur. This may be phosphorylation of eNOS by CaMKII or by AMPK [104], [105] which is in turn activated by Ca-calmodulin dependent kinase kinase (CaMKK) [106], or even an activation of PKB by CaMKII [107], [108].

### Possible Roles of Long PFL(3) in Ca^2+^ Signalling System

It is relatively unlikely that the action of long PFL(3), which may have a long time delay upon sequential activation of different enzymes of this pathway (Fig. 5), could cause the observed fast [Ca^2+^]_i_ oscillations with a period of several dozens of seconds (Fig. 1).

This long PFL(3) from a theoretical point of view could perform several functions in the system:

1. it may serve as a source of slow Ca^2+^ oscillations with long periods and high amplitudes;
2. by setting the level of cADPR, it may provide the necessary conditions required for the generation of fast Ca^2+^ oscillations by short PFL(1) i.e. CICR from RyR (where cADPR serves as a co-agonist of Ca^2+^ or a modulator of RyR activity) [76];
3. it may generate complex multiperiodic or irregular shaped (chaotic) oscillations due to interplay with the short PFLs (1,2) and create the properties of multistationarity in the system and the switching phenomena;
4. it may enable the switching off of the chain G_αq_→PLC→IP_3_→Ca^2+^ by inhibiting IP_3_R upon phosphorylation of IRAG by PKG and inhibiting PLC upon phosphorylation of RGS proteins with the participation of PKG;
5. it may participate in the control of the activities of Ca^2+^-ATPases and different Ca^2+^-channels upon their phosphorylation by PKG, thus creating new NFLs important in the control of [Ca^2+^]_i_ level.

### The Parametric Modulation of Long PFL(3) by Intermediates Inserted in it

The parametric modulation of activity of the long PFL(3) occurs naturally with the implication of the signalling axis G_βγ_→PI3Kγ→PKB by ACh, leading to activation of eNOS upon its phosphorylation by PKB. The intermediates of this long PFL(3) (NO, cGMP, cADPR), upon corresponding addition of an NO donor (SNAP), a permeable analog of cGMP (8-Br-cGMP) or a substrate of ADP-ribosyl cyclase (ADPRC) or CD38 (βNAD) may modulate the Ca^2+^ oscillations generated by short PFL(1) or may produce the oscillations on their own. In this way we can determine the role of PFL(3) in the generation of fast Ca^2+^ oscillations.

If the signalling loop intermediate added in the incubation medium quickly enters the cells and equilibrium is quickly established, this means that clamping (fixation) of the variable of PFL can occur. In this case the long PFL(3) would break and the possible sources of oscillations could only be related to CICR via RyR, or CICR via IP_3_R, or some other mechanism.

If, on the other hand, an intermediate of the signalling loop slowly penetrates into the cell, and the influx has nearly constant speed, this would not break the long PFL, but would parametrically modulate its activity. Slow release of NO from SNAP may model influx of NO into the cell (input). Activation of PKG by 8-Br-cGMP or substrate control of ADPRC (or CD38) by βNAD, may represent modulation of the corresponding enzyme activities.

**Figure 8:**
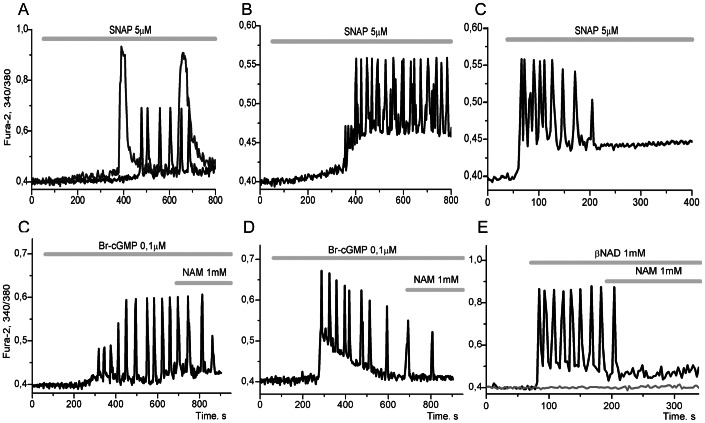
The induction of various modes of [Ca^2+^]_i_ oscillations in adipocytes by the intermediates of long PFL(3). **A**: upon application of 5 µM SNAP, a donor of NO, the generation of fast Ca^2+^ oscillations occurs in 34±9,6% of the cells (black curve) and slow high-amplitude Ca^2+^ oscillations (blue curve) are observed in 11±2,5% of adipocytes, (n = 208). **B**: the transition of the system to a steady oscillatory mode with a high mean value of Ca^2+^ for the oscillation period in 15±3,6% of the cells, (n = 97). **C**: the transition of the system to a new stable stationary state with a high level of calcium in 59±9,8% of the cells, (n = 105). **D**: [Ca^2+^]_i_ oscillations with a period of several dozen seconds are induced in 43±6,1% of the cells by the application of 0,1 µM 8Br-cGMP, a permeating analog of cGMP, and are quickly suppressed by an inhibitor of ADPRC – Nicotinamide (NAM), (n = 132). **E**: [Ca^2+^]_i_ oscillations with varying period of oscillations are induced in 26±11,0% of adipocytes by the application of 0.1 µM 8Br-cGMP and are quickly suppressed by NAM, (n = 116). **F**: oscillations caused by the application of 1 µM βNAD are observed in 57±10,3% of adipocytes and are quickly suppressed by nicotinamide (NAM) (n = 163). There are no [Ca^2+^]_i_ oscillations in 43±8,6% of the cells (grey curve).

Figure 8 shows typical examples of various types of steady oscillations or transit modes, arising in separate cells upon parametric manipulations by the application of sources of NO, cGMP or cADPR to the incubation medium of adipocytes.

In the absence of ACh, addition into incubation medium of SNAP (Fig. 8A–C; input of NO into long PFL), 8-Brc-GMP (Fig. 8D, E; modulation of PKG activity) or βNAD (Fig. 8F; modulation of ADPRC (or CD38) activities) induces following set of dynamic regimes:

- steady fast periodic regimes (fast Ca^2+^ oscillation) with periods ranging from several seconds to several dozens of seconds (Fig. 8B, D, F);
- steady slow spike-like periodic regimes, characterized by high Ca^2+^ amplitudes and long period lengths of several minutes or more (Fig. 8A, blue curve);
- switching phenomena with transitions of Ca^2+^ signalling system to a new stationary state:
- to stable *stationary state (SST) with a higher level of [Ca^2+^]_i_* (Fig. 8C);
- to steady *periodic regime with high mean value of [Ca^2+^]_i_* for oscillation period (Fig. 8B).

Figure 8B may correspond to hard Hopf bifurcation or hard excitation (after Minorsky, 1962) and may represent the transition from stable focus or node (SST) to stable limit cycle (unstable focus, see for example [41], [69]).

Figure 8C may correspond to switching from SST stable focus type or node to another SST of stable focus type.

Application of SNAP or 8-Br-cGMP promotes different dynamic regimes after a short time delay (Fig. 8 A–E) in comparison with the fast immediate action of βNAD (Fig. 8 E). All the registered modes of Ca^2+^-oscillations disappear upon the addition into the incubation medium of nicotinamide (NAM), a product and inhibitor of ADPRC (Fig. 8, D–F), or ryanodine, an inhibitor of RyR (not shown).

However, the retention of fast modes of Ca^2+^-oscillations in the presence of SNAP, Br-cGMP and βNAD indicates that in all these regimes the long PFL(3) acts as a modulator of the fast oscillatory mechanism based on CICR via RyR (on short PFL(1)), providing the required level of cADPR, a coagonist of Ca^2+^ at the RyR. The exceptions are slow periodic regimes presented on figure 8A (blue curve) and figure 7. In these regimes the long PFL(3) apparently serves as the generator of slow oscillations.

Switching phenomena (Fig. 5G, H; Fig. 8B, C) may also reflect the property of multistability in the Ca^2+^- signalling system, i.e. the property of coexistence of different dynamic regimes in this system. This coexistence of SST and periodic or chaotic regimes is a well-known property of non-linear dissipative systems, as follows from mathematical modeling [42], [66]–[68].

### The Parametric Modulation of Operation of Long PFL(3) by Atrial Natriuretic Peptide (ANP)

**Figure 9:**
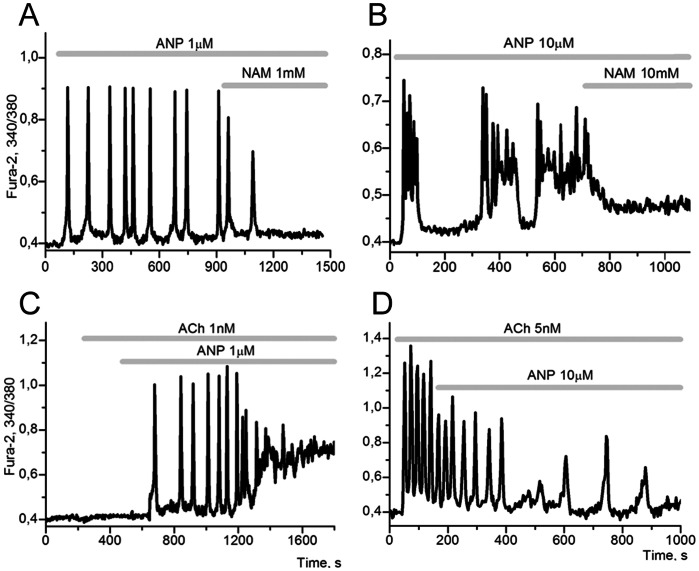
The onset of different modes of Ca^2+^ oscillations in adipocytes under the influence of ANP. **A**: the application of 1 µM ANP results in the generation of fast Ca^2+^ oscillations in 38±10,5% of adipocytes. The addition of 1 mM nicotinamide (NAM) suppresses the periodic modes in all cells, (n = 272). **B**: the application of 10 µM ANP can, in addition to the typical fast [Ca^2+^]_i_ oscillations, cause stochastic (chaotic) oscillations in 5±4,2% of adipocytes which are also suppressed with the addition of NAM, (n = 179). **C**: the effect of potentiation of 1 nM ACh with 1 µM ANP is observed in 43±10,7% of adipocytes, (n = 113). **D**: the application of 10 µM ANP against the background of oscillations induced by 5 µM ACh results in suppression of Ca^2+^ oscillations in 7±2,2% of adipocytes, (n = 154). The majority of cells do not demonstrate the suppression of oscillations.

Application of atrial natriuretic peptide (ANP), a direct activator of membrane guanylate cyclase (mGC), triggers the input of cGMP into the long PFL(3). Such input can leads to a wide range of dynamic effects on the system: the generation of fast Ca^2+^ oscillations containing a single modal value (Fig. 9A); chaotic Ca^2+^ oscillations (like bursting with varying periods and amplitudes (Fig. 9B); transition of the system from SST with a low Ca^2+^ level to SST with a higher Ca^2+^ level (Fig. 9C). Upon application of ANP a short lag-period occurs before the onset of oscillations, similar to that observed for SNAP or 8-Br-cGMP (Fig. 8).

All the described modes, occurring in the presence of ANP, also disappear after application of inhibitors of RyR (not shown) or ADPRC (1 mM NAM; Fig. 9A, B).

Like NE, low concentrations of ANP can also potentiate the effect of low doses Ach (not shown) and higher (about 1 µM) doses ANP produce bursts of Ca^2+^ oscillations with subsequent transition to a new SST (Fig. 9C). Upon increasing the concentrations of ACh and ANP, the effect of ANP can result in suppression of Ca^2+^ oscillations promoted by ACh (Fig. 9D). Such switching off of ACh signalling may be related to accelerated Ca^2+^ extrusion due to an over activation of Ca^2+^ ATPases by PKG, i.e. due to an amplification of NFLs.

This later proposal may be supported by results previously obtained on different cells. It was shown earlier that ANP diminished Ca^2+^ signals, evoked in endothelial cells, and smooth muscle cells presumably by activating Ca^2+^ removal processes by PKG [109], [110].

## Discussion

Currently very little is known about the role of Ca^2+^ and Ca^2+^ signalling systems in the regulation of lipid metabolism in WAT [111], [112], although patented methods involving sequential treatment of patients with hyper- and hypocalcemic diets have been shown to produce significant WAT mass reduction [113]. This implies that Ca^2+^ may play an important role in the control of WAT lipid metabolism, and that lipid metabolism, in the state of obesity, is fragile with respect to drastic perturbations in Ca^2+^ influx. In this context the analysis of the mechanisms underlying dynamic behavior of the Ca^2+^ signalling systems in adipocytes may be a very important tool in the further theoretical and experimental studies of the mechanisms of autoregulation of this system and of Ca^2+^ control of metabolic systems in adipocytes under normal conditions and in different pathological stages, such as the adipose tissue dysfunction [114] and adipocytes hypertrophy and death [115], [116] in type 2 diabetes.

Taken together, the results presented above suggest that observed complex dynamic regimes (the periodic and chaotic oscillatory regimes, the generation of Ca^2+^ impulses and the switching phenomena in the system of Ca^2+^ signalling of WAT adipocytes) represents an intrinsic fundamental property of this system.

These results indicate that in rodent adipocytes the effect of ACh is realized through M_3_-AChRs and results either in the generation of trains of Ca^2+^ impulses with possible receptor desensitization, or in the generation of sustained Ca^2+^-oscillations (Fig. 1). At low concentrations of ACh, which are unable to provoke notable Ca^2+^ responses in most adipocytes, the subsequent activation of α_1_- or α_2_-adrenergic receptors leads to potentiation of the effect of ACh and induces different dynamic regimes, including Ca^2+^-oscillations or switching phenomena (Fig. 4). The observed effects disappear upon incubation of cells with PTX, an inhibitor of G_q_ and G_i_ proteins (Fig. 2B), with inhibitors of PI3K (Fig. 3A, B), PKB (Fig. 3C), or eNOS (Fig. 3D). This indicates that there is a convergence of signalling pathways involving M_3_-cholinergic and α_1_- and/or α_2_-adrenergic receptors, which is connected with the effects of G_βγ_-subunits of G_q_ proteins (for M_3_- and α_1_-receptors) and G_i_ proteins (for α_2_-receptors) on PI3Kγ, leading to the activation of a signalling axis:

G_βγ_→PI3Kγ→PKB→eNOS.

This signalling axis activates eNOS and parametrically modulates the functioning of long PFL(3).

A second signalling axis, participating in parametric control of long PFL(3), is realized as result of ANP action and represents an input of cGMP into this loop:

G_α_→mGC→cGMP→PKG.

In addition to these signalling axes, a third axis participating in parametric control of long PFL(3) may be realized via different Ca^2+^ influx pathways focused on CaMKII, with consequent phosphorylation of eNOS, RyR, IP_3_R etc, while a fourth may be realized via β-adrenoreceptors and the cAMP/protein kinase A (PKA) dependent pathway. Future study of the roles of these axes may provide further insights but is beyond the scope of the present work.

What could the physiological relevance of the potentiating effect of NE on ACh action be? It implies that even if the parasympathetic nervous system poorly innervates different WAT depots, the low doses of ACh transported by blood may, in combination with epinephrine, produce the same parametric control of long PFL(3) as higher doses of ACh can by itself (Fig.1,4). The same may also be true for the activating effect of low doses of circulatory ANP (Fig. 9B).

In adipocytes, as well as in cells of other types [31], [36], [37], Ca^2+^-waves are observed upon the application of ACh, ANP, CCK, BK and other agonists (not shown). The appearance of Ca^2+^-oscillations and the propagation of Ca^2+^-waves in endocrine and smooth muscle cells are usually explained by the realization of the CICR mechanism for IP_3_R [6], [30]–[34], [58] or by the formation of PFL upon activation of PLC by Ca^2+^ [6], [59]–[62], less frequently by CICR with the involvement of RyR [12], [14], [35].

Using our experimental conditions (i.e. with incubation media containing L-arginine), the mechanisms of oscillations and the active conducting medium for wave propagation are coupled to RyR, with its short and long PFLs. Active interplay between long PFL(3) and short PFL(1), based on CICR via RyR, brings about the complex dynamic behaviour observed (Fig. 4, 7–9).

The persistence of Ca^2+^-oscillations in adipocytes in the presence of PLC, IP_3_R and PKC inhibitors (Fig. 2), indicates that IP_3_R inhibition (upon its possible phosphorylation by PKG) and switching off of the entire signalling chain (A) can be realized in our experimental conditions.

It may be assumed that activation of IP_3_R in the initial moment of ACh action may play the role of a “spark plug” for activation of RyR or eNOS (i.e. of the long PFL(3)), providing the level of [Ca^2+^]_i_ required for activation of RyR or eNOS, until the moment of complete activation of the long PFL(3). Tandem mechanisms implying a potentiating action of IP_3_R on RyR have been proposed by several authors [37]–[39]. However, the induction of Ca^2+^-oscillations by ACh in the presence of PLC, IP_3_R and PKC inhibitors allows us to consider such a possible mechanism of action for the signalling pathway (A) as secondary or unlikely under our conditions.

A phase shift between the maxima of Ca^2+^ and NO oscillations, observed at slow periodic regimes (Fig. 7), indicates that in addition to fast activation of eNOS by Ca^2+^ (by CaCaM) some slower Ca^2+^-dependent processes may also participate in the reinforcement of long PFL(3).

It is well known that the activity of eNOS is strongly dependent on the [Ca^2+^]_i_ level and on the phosphorylation of eNOS by kinases of different types. The enzyme is activated by the CaCaM complex [76], [77]. Ca^2+^-dependent CaMKII takes part, along with PKB, PKA, AMPK and PKG, in the covalent modification and activation of this enzyme [104], [105]. However, it is currently unknown whether the activation of eNOS by PKG is realized in the cell, and the efficiency of its activation with CaMKII and AMPK remains uncertain.

As a result of such multiple regulations of eNOS besides long PFL(3) and short PFLs (1,2), several additional long PFLs can be formed:

eNOS→NO→cGC→cGMP→PKG→eNOS (4)

Ca^2+^→CaMKII→eNOS→NO→sGC→cGMP→PKG→CD38→cADPR→RyR→Ca^2+^, (5)

Ca^2+^→CaMKK→AMPK→eNOS→NO→sGC→cGMP→PKG→CD38→cADPR→RyR→Ca^2+^, (6)

Furthermore, it is known that NO and cGMP may bring about the phosphorylation of PKB by CaMKII [107], [108], allowing for the possible functioning of yet another long PFL(7):

Ca^2+^→CaMKII→PKB→eNOS→NO→sGC→cGMP→PKG→CD38→cADPR→RyR→Ca^2+^ (7)

**Figure 10:**
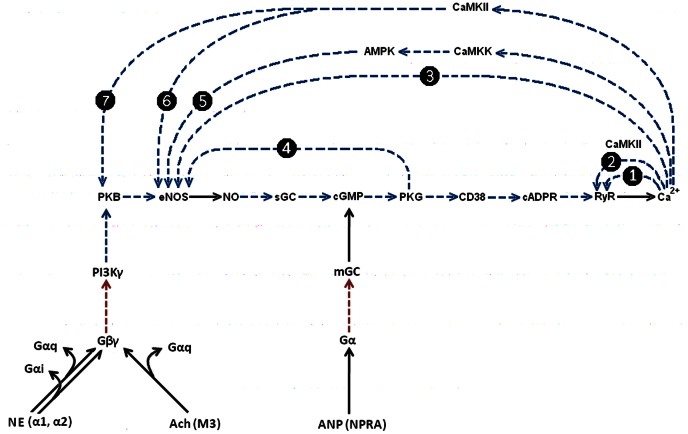
Multivariable multi feedback network with a family of nested PFLs, controlling Ca^2+^-signalling pathway (B) and integrating the signals from ACh, NE and ANP. Dashed blue numbered arrows indicate various PFLs operating in the system. Modulating axes, activating PKB and PKG are also presented. For details see the text.

All these PFLs are presented in figure 10 and shown by numbered arrows (in blue). Signaling axes parametrically modulating this multiloop network and integrating the signals from ACh, NE and ANP also presented on this chart.

Thus, short PFLs (1, 2) and long PKG-dependent PFL(4) are inserted into long PFL(3). CaMKII, AMPK and CaMKK dependent PFLs (5, 6, 7) embrace all above mentioned loops.

This multivariable multi feedback network with a family of nested PFLs controlling Ca^2+^ signalling pathway (B) may be considered as a kinetic model of Ca^2+^ signalling via RyR in adipocytes.

So, we have a family of nested PFLs. Such multiloop control is not excessive. The redundancy, created by each new loop, may reinforce the operation of main PFL (3) and increase the reliability of a multiloop system. This reliability may however bring about some difficulties in experimental studies intended to evaluate the weight and role of these different loops. Of course, this claim on the reliability of this multivariable multiloop network remains to be proven by numerical studies.

It is known from automatic control theory [117], [118] that NFLs may: increase stability of the system; reduce the system response to noise; and improve the robustness of the system, i.e. functional stability against parameter variation. The systems with common NFLs, embracing all unreliable elements (i.e. systems with multiloop control) achieve this more effectively than systems with local NFLs.

Cellular aerobic energy metabolism, with its family of nested common and local NFLs, may be considered as an example of such a system. Low sensitivity of fluxes to variation of different parameters in this system may represent an example of functional robustness in energy metabolism. The appearance of negative feedforward loops (dynamically equivalent to PFLs) in some pathological states (in some inborn errors) may bring about to collapse of its functioning [119].

In modern systems biology the robustness has many new features [120]–[123] and is mainly based on the operation and redundancy of NFLs [121], [123], although in some models a combination of both negative and positive feedback loops can also contribute to system robustness [121]–[125].

PFLs may increase the sensitivity of dynamic systems [124], [125]. In general, PFLs and negative feedforward loops may create multistability and hysteresis, oscillations, waves, etc [41], [69], [126], [127]. On the other hand, PFLs may also destroy robustness and introduce fragility [128]. In this context, fragility is undesirable property of dynamical system.

The Ca^2+^-signalling system considered above, with its family of nested PFLs (Fig. 10), is extremely sensitive to changes of input parameters, determined by combinations of agonists used. It responds by spikes, oscillations or switching phenomena with enlarged steady or oscillating Ca^2+^-level in the cytosol (Fig. 4, 9). This extremely sensitive system is not fragile and maintains the ability to offer a set of spatiotemporal patterns in response to different combinations of agonists. In this context, the Ca^2+^-signalling system based on family of nested PFLs may be characterized as “sensitive but robust”. The reliability and the robustness of its operation is apparently based on the nested structure of PFLs in the network (Fig. 10), on redundancy and reinforcement of the operation of the main PFL(3) by other PFLs, and also on the operation of several NFLs.

Under the conditions of the presented experiments we cannot evaluate the role of the PFL(4) with the participation of PKG. This enzyme plays the role of house-keeping enzyme in the cell, controlling Ca^2+^ release from IP_3_R [79]–[82] and RyR (via PFL(3)), Ca^2+^ extrusion by Ca^2+^-ATPases [100]–[103], influx of Ca^2+^ via various channels [129]–[132] and also participates in desensitization of effects of different agonists through RGS proteins [83], [84].

The preliminary studies indicate that the PFLs implicating CaMKII (5, 6, 7) may be involved but are not critical under action of ACh. Further experiments are required to clarify the role of CaMKII in the Ca^2+^-oscillations in response to ACh. Although not essential for the action of ACh alone, the role of CaMKII as well as AMPK and CaMKK may be significant under the potentiating action of ANP, and perhaps even more so in response to CCK and BK with their modulatory effects on PFL(3) via Ca^2+^-influx pathways.

Therefore, to explain the effects mentioned above (presented in Fig. 1, 4, 5, 7, 9) one can consider the main PFL to be long PFL(3), with supporting roles for the other long PFLs with the participation of PKG (4), AMPK (5), and CaMKII (6, 7).

**Figure 11:**
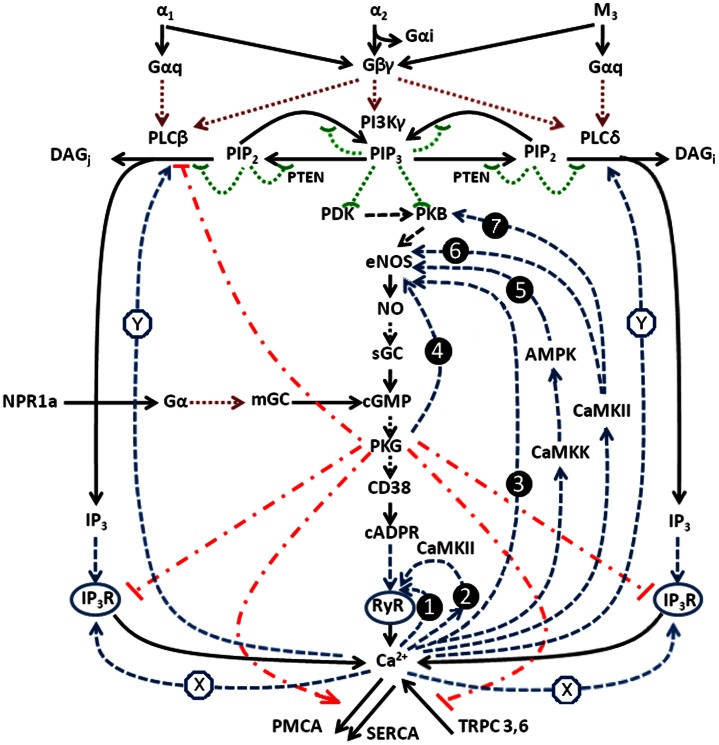
Total kinetic model of Ca^2+^ signalling in adipopocytes with participation of Ca^2+^ signalling pathway (A) and (B). Various types of activation are shown by dashed arrows or dashed lines with the icon (⌢). Various types of inhibition are shown by dashed stroke with the icon (T). Formed PFLs are shown in blue and NFLs in red. Numbered PFLs operating in the pathway (B) correspond to those on Fig. 10. PFLs operating in the pathway are indicated by symbols X and Y in the circles. The description of the schematic is given in the text.

The general kinetic model of Ca^2+^ signalling in adipocytes upon the activation of M_3_-cholinergic and α-adrenergic receptors and NPRA may be presented as follows (Fig. 11). In this schematic, dashed arrows and dashed lines with the icon (⌢) represent various types of activation for enzymes of different signalling pathways. The dashed stroke with the icon (T) indicates inhibition.

As can be seen in the diagram, Ca^2+^-dependent activation of Ca^2+^-release (CICR) from IP_3_R and RyR, with the modulating influence of co-agonists IP_3_ and cADPR respectively, forms two short PFLs in the system, indicated by symbols X and 1 in the circles. The main long PFL(3) arises from the activation of eNOS with Ca^2+^. Other long PFLs are formed with participation of CaMKII_β_, AMPK, CaMKK, PKB and PKG. All these PFLs are shown by numbered circles in blue. Positive feedback arising from activation of PLC by Ca^2+^ is also indicated in blue by the symbol Y in the circle.

Meanwhile, NFLs acting to stabilize the Ca^2+^ level are formed through: the activation of Ca^2+^-ATPases upon their phosphorylation by PKG; phosphoinhibition of IP3R (via IRAG) and PLC (via RGS); the inhibition by PKG of various Ca^2+^ channels representing Ca^2+^ influx pathways. All these NFLs are shown in red.

The system of reactions of phosphoinositide metabolism also forms several positive feedback loops (dashed lines with the icon (⌢)), based on the possible domain (pH, pX, etc.) activation of PI3K and phosphatase and tensin homolog (PTEN) by their products phosphatidylinositol (3, 4, 5) trisphosphate (PIP_3_) and phosphatidylinositol (4, 5) bisphosphate (PIP_2_). The role of such “autocatalytic activation” is currently relatively poorly studied, although it may be very important in the convergence of signals coming from different receptors with the participation of G_αq_ and G_βγ_ proteins and tyrosine kinases (TK).

The chart also includes two signalling axes modulating the primary PFL(3):

M_3_, α_1_, α_2_-receptors→G_βγ_→PI3Kγ→PDK→PKB→eNOS.

NPRA→G_α_→mGC→cGMP→PKG,

These axes provide the parametric control of the long PFL(3) by activation of eNOS and PKG respectively.

The modulation by ANP of the long PFL(3) activated by G_βγ_ proteins, can: promote the generation of Ca^2+^-oscillations and switching phenomena (Fig. 9A-D), potentiate the effect of low doses of ACh (Fig. 9C) or suppress the effect of ACh due to the activation of Ca^2+^ extrusion by Ca^2+^ ATPases at high PKG activity (Fig. 9D).

And “vice versa”, the same effects we have to expect for modulation of PFL(3), activated by ANP by entry from G_βγ_ proteins side.

In this way we may have crosstalk between signalling axes at the level of whole long PFL(3).

The presented data indicate that the long PFL(3), reinforced by other PFLs, can potentially fulfill several functions in the Ca^2+^ signalling system in adipocytes:

1. it may serve as a source of Ca^2+^-oscillations of long period and high amplitude;
2. by controlling the levels of cADPR, a co-agonist of Ca^2+^ at the RyR, it may provide the necessary conditions for the generation of fast Ca^2+^ oscillations with the implication of a short PFL of the mechanism of Ca^2+^- induced Ca^2+^ release (CICR) at the RyR;
3. it may generate complex or irregular shaped Ca^2+^ oscillations due to its interplay with short PFL(1) and also create the properties of multistationarity and switching phenomena;
4. it may control the activity of PKG and thereby control the activities of Ca^2+^-ATPases and Ca^2+^-channels which are modulated by phosphorylation by PKG, thus creating negative feedback loops and participating in the control of [Ca^2+^]_i_ level;
5. it may act to switch off the signalling chain G_αq_→PLC→IP_3_→Ca^2+^ by inhibiting IP_3_R upon phosphorylation of IRAG by PKG and inhibiting PLC upon phosphorylation of RGS proteins with the participation of PKG.

Point (5) assumes that integrated, non-selective (promiscuous) signalling via G_βγ_ proteins may predominate over selective G_αq_ signalling by suppressing the IP_3_R functioning (via PKG) and dissociating Ca^2+^ and DAG/IP_3_ (IP_4_, IP_5_) signals in the cells.

### Conclusions

In conclusion, the model of Ca^2+^-signalling system (Fig. 11) presented in this study represents a multiloop nested feedback network constructed of a family of nested PFLs and several NFLs. This network may be considered a sensitive Ca^2+^-controller in the cell, which responds to integrated action of various agonist with a set of spatiotemporal patterns, either with subsets of states with: spikes, fast or slow Ca^2+^-oscillations or by switching of the system to the state(s) with enlarged Ca^2+^-level (stable or average). Considering the parasympathetic control of adipose tissue and a possible role for Ca^2+^ in the regulation of lipid metabolism, the very complex organization of Ca^2+^ signalling system may have important implications for the function of this endocrine tissue.

## Supporting Information

Figure S1 (DOCX)
