# Supplementary material for: Acetylcholine Promotes Ca2+and NO-Oscillations in Adipocytes Implicating Ca2+→NO→cGMP→cADP-ribose→Ca2+ Positive Feedback Loop - Modulatory Effects of Norepinephrine and Atrial Natriuretic Peptide
Source: PLoS One. 2013 May 16;8(5):e63483. doi: 10.1371/journal.pone.0063483 (PMC3656004; doi:10.1371/journal.pone.0063483)
Supplement: Figure S1 — (DOCX) [file pone.0063483.s001.docx]

**Figure S1.** **The culture of white mouse adipocytes.**

The culture age is 9 DIV; 20-fold magnitude. **А** – Bright field microscopy; **B** – Fura-2 fluorescence upon excitation at 380 nm.

| 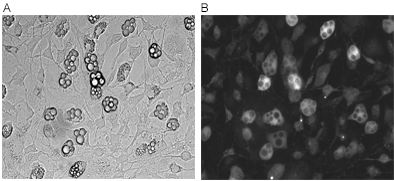 |
| --- |

Supplementary Figure S1
